# Expression of concern: Highly stereoselective gram scale synthesis of all the four diastereoisomers of Boc-protected 4-methylproline carboxylates

**DOI:** 10.1039/d3ra90061d

**Published:** 2023-07-07

**Authors:** Kehuan Sun, Cheng Tao, Bohua Long, Xiaobin Zeng, Zhengzhi Wu, Ronghua Zhang

**Affiliations:** a College of Traditional Chinese Medicine, Jinan University Guangzhou 510632 China; b Integrated Chinese and Western Medicine Postdoctoral Research Station, Jinan University Guangzhou 510632 China; c The First Affiliated Hospital of Shenzhen University Shenzhen 518035 China bhlong121@163.com; d Shenzhen Institute of Geriatric Medicine Shenzhen China; e Central Center Lab of Longhua Branch, Department of Infectious Disease, Shenzhen People's Hospital, 2nd Clinical Medical College of Jinan University Shenzhen 518120 Guangdong Province China

## Abstract

Expression of concern for ‘Highly stereoselective gram scale synthesis of all the four diastereoisomers of Boc-protected 4-methylproline carboxylates’ by Kehuan Sun *et al.*, *RSC Adv.*, 2019, **9**, 32017–32020, https://doi.org/10.1039/C9RA06827A.

The Royal Society of Chemistry is publishing this expression of concern in order to alert readers that concerns have been raised regarding the integrity of the NMR data in the ESI.

Several impurity peaks have been removed from the NMR spectra. The authors have provided the original raw data, however, an independent expert has stated it would not be appropriate to re-publish the original spectra and requested the authors reproduce their results.

An expression of concern will continue to be associated with the article until we receive conclusive evidence regarding the reliability of the reported data.

Laura Fisher

30th June 2023

Executive Editor, *RSC Advances*

## Supplementary Material

